# The Impact of Physical Form on the Biocompatibility of Poly(3-hexylthiophene-2,5-diyl)

**DOI:** 10.3390/ma18204671

**Published:** 2025-10-11

**Authors:** Daniela A. Tudor, Sorin David, Mihaela Gheorghiu, Szilveszter Gáspár

**Affiliations:** 1 International Centre of Biodynamics, 1B Intrarea Portocalelor, 060101 Bucharest, Romania; dtudor@biodyn.ro (D.A.T.); mgheorghiu@biodyn.ro (M.G.); 2 Faculty of Biology, University of Bucharest, 91-95 Splaiul Independenței, 050095 Bucharest, Romania

**Keywords:** P3HT films, P3HT nanoparticles, P3HT biocompatibility, photoexcitation of P3HT, electrically conductive polymers, endothelial cells

## Abstract

Poly(3-hexylthiophene-2,5-diyl) (P3HT) is a semiconducting, electron donor polymer which, in addition to its intensive use in optoelectronic devices, is increasingly investigated in biological systems. However, there are conflicting reports about the biocompatibility of P3HT, and no direct comparison between P3HT films and P3HT nanoparticles has been conducted. In this context, we investigated the viability of bEnd.3 endothelial cells when such cells are grown onto P3HT films or incubated with P3HT nanoparticles and subjected to trains of moderate power density, relatively long light pulses. We observed that, while P3HT films do not decrease the viability of bEnd.3 cells at all, P3HT nanoparticles lower the viability of bEND.3 cells by ~20%, when the nanoparticles also contain [6,6]-phenyl-C61-butyric acid methyl ester (PCBM) as electron acceptor, and by ~30%, when the nanoparticles do not contain PCBM. Interestingly, the used photoexcitation protocol did not impact the biocompatibility of the P3HT-based materials. The obtained results reveal that (i). nanostructuring has a detrimental impact on the compatibility of P3HT with bEND.3 endothelial cells, and (ii). P3HT-based materials can be safely combined with light when used in biological systems because light, as used in the present study, does not alter the biocompatibility of such materials.

## 1. Introduction

Poly(3-hexylthiophene-2,5-diyl) (P3HT) is an electron donor (i.e., p-type), semiconducting polymer. Because of its intense use in organic photovoltaic devices, P3HT was described as “a workhorse among conjugated semiconducting polymers” [1] and also as “the fruit fly among polymeric organic semiconductors” [2]. The combination of P3HT with [6,6]-phenyl-C61-butyric acid methyl ester (PCBM) is also very popular, hence this combination of electron donor and electron acceptor materials was described as “best seller in polymer photovoltaic research” [3]. In addition to this massive use of P3HT in the development of organic photovoltaic devices, P3HT has also found interesting biomedical applications. These applications rely not only on the photoexcitability of P3HT but also demand that this polymer has a good biocompatibility (before, during and after photoexcitation). The biomedical applications of P3HT films, fibers and particles can be divided into the following four groups: (i). use of P3HT in cellular differentiation [4,5,6,7,8,9,10,11], (ii). use of P3HT in tissue engineering [12], (iii). use of P3HT for stimulation/inhibition of cells [13,14,15,16,17,18,19,20], and (iv). use of P3HT in redox therapy [21,22,23,24]. It must be noted that there are important overlaps in between these four uses of P3HT films and nanoparticles. For example, the generation of reactive oxygen species (ROS) by P3HT-based materials clearly plays an important role not only in redox therapy but also in the differentiation of cells with P3HT-based materials and in tissue engineering with P3HT-based materials. That is simply because ROS were shown to impact those cellular processes (see, for example, Refs. [25,26]). It is also worth noting that using P3HT to modulate the electrical activity of cells is among the polymer’s most advanced biological applications: P3HT films and nanoparticles have already been used to restore light sensitivity in the retinas of rat models of Retinitis Pigmentosa [17,18,19,20].

When going through the literature describing the biomedical applications of P3HT, one notes the following provocative facts:

(i). The biocompatibility of P3HT remains somewhat unclear because of several reasons; For example, many studies on the biocompatibility of P3HT use P3HT after oxidizing it (e.g., with an oxygen plasma treatment [4,14] or with Rozen’s reagent [16]), after mixing it with other materials (e.g., with other polymers [9,10,12,23,24,27], carbon nanotubes [28], etc.) or after coating it with other materials (e.g., with fibronectin [6,11,15,21,29,30], polylysine [9,13,28,31,32], polypyrrole [9], etc.). As a consequence, the biocompatibility (or the lack of it) observed in these studies is not entirely that of P3HT. When complications brought about by oxidation, mixing and/or coating of P3HT are not present, the literature on the biocompatibility of P3HT is still contradictory. Some mouse fibroblasts (e.g., L929) were observed to not adhere to and grow onto P3HT films at all [33]. Other mouse fibroblasts (e.g., 3T3) were observed to adhere to and grow onto P3HT films but to show significantly lower viability than on plastic cell culture ware [34]. Roughly 30% of PC-12 cells (originating from rat adrenal pheochromocytoma) died when cultured on P3HT-based nanofibers subjected to photoexcitation and only coating these nanofibers with polypyrrole lowered the number of dead cells [9]. The compatibility of P3HT with human embryonic kidney 293 cells (HEK-293 cells) remains a subject of debate as well. Some studies indicated that P3HT films [30] and P3HT nanoparticles [35] do not affect the viability of HEK-293 cells. Other studies [36,37] show that HEK-293 cells incubated with P3HT nanoparticles exhibit a 20–40% reduction in viability compared to untreated cells. Factors such as the specific cellular model, the way P3HT is presented to the cellular model, and the exact manner P3HT is photoexcited play critical roles in determining the biocompatibility of P3HT. Therefore, the biocompatibility of P3HT-based materials cannot be assumed and requires thorough investigation.
(ii). While an important advantage of P3HT (in comparison with other polymers) is the photoexcitability of this polymer, there are many studies on the biocompatibility of P3HT which were carried out in the absence of a controlled illumination (e.g., [5,12,27,28,33,34,36]). Moreover, when controlled illumination was used, the parameters of the illumination were quite different from one study to the other and not always properly justified. For example, Aziz et al. photoexcited P3HT in contact with human adipose tissue-derived stem cells (hASCs) using light with a wavelength of λ = 532 nm, light power density of LPD = 20 mW/cm^2^ and using 20 ms (or 400 ms) long pulses distanced at 200 ms (or 4000 ms) from each other [4]. Meanwhile, Lodola et al. photoexcited P3HT in contact with endothelial colony-forming cells (ECFCs) using light with λ = 525 nm, LPD = 40 mW/cm^2^ and using 30 ms long pulses distanced at 70 ms from each other [6]. Criado-Gonzalez et al. photoexcited nanoporous P3HT thin films in contact with human umbilical vein endothelial cells (HUVECs) with a light with λ = 520 nm, LPD = 110 mW/cm^2^ and using a 2.5 s long pulse [21]. Interestingly enough, somewhat smaller power density (i.e., 6 mW/cm^2^) was used to generate ROS with porous P3HT nanoparticles internalized by HUVEC cells [22]. Most of the used LPDs exceed those we experience during normal activities and are far from being comfortable for human eye. They also come with important heat dissipation that explains the short duration of the light pulses used during the investigations.
(iii). In addition to this, a thorough, side-by-side comparison of the biocompatibility of P3HT films and the biocompatibility of P3HT nanoparticles made of the same polymer batch is also missing, although it is widely accepted that the biocompatibility of a material could show dependency on the lateral size of the material [38,39].

In this context, our aim was to comparatively investigate the biocompatibility of P3HT films and P3HT nanoparticles (synthesized from the same P3HT batch, with or without PCBM) when these films and nanoparticles are photoexcited with a LPD similar to that we normally encounter in office areas and outdoor on a moderately sunny day (more specifically, 1.3 mW/cm^2^). It was also our aim to prolong the duration of the illumination when testing the biocompatibility of P3HT (from the most often used ms range to the s range) to bridge in vitro and in vivo conditions of use. Because of being obtained in such conditions, the findings described below are most relevant for the use of P3HT in restoring the light sensitivity of retina (as shown in Refs. [17,19]).

## 2. Materials and Methods

### 2.1. Materials

Regioregular P3HT (catalog no. M1010, with molecular weight of 74,000 g/mol) and PCBM (catalog no. M111, >99%) were purchased from Ossila Ltd. (Sheffield, UK). Chloroform (catalog no. C2432), chlorobenzene (catalog no. 284513), dimethyl sulfoxide (catalog no. D4540, ≥ 99.5%), sodium dodecyl sulfate (SDS, catalog no. L4390), potassium phosphate monobasic (catalog no. P5655), potassium chloride (catalog no. P9333), sodium chloride (catalog no. 31434), di-sodium hydrogen phosphate (catalog no. 1.06585), trypsin-EDTA solution 10x (catalog no. 59418C) and fetal bovine serum (FBS, catalog no. F7524) were purchased from Sigma-Aldrich Co. (Saint Louis, MO, USA). 3-(4,5-dimethylthiazol-2-yl)-2,5-diphenyltetrazolium bromide (MTT, ≥98%, catalog no. 4022.1) was purchased from Carl Roth GmbH (Karlsruhe, Germany). All culture media and supplements used for cell culturing were purchased from Thermo Fisher Scientific Inc. (Waltham, MA, USA) and Sigma-Aldrich Co. All chemicals were used as received if not otherwise stated. All solutions were prepared using ultrapure water from a Direct-Q 3 UV water purification system (from Millipore SAS, Molsheim, France) if not otherwise stated.

### 2.2. Synthesis of the P3HT Nanoparticles and P3HT-PCBM Nanoparticles

P3HT nanoparticles were synthesized using a procedure adapted from literature [40,41]. In line with the respective procedure, 13 mg P3HT polymer was dissolved in 1 mL chloroform and then added into 5 mL of aqueous SDS solution (5.8 mg/mL). A “macro-emulsion” was first prepared by stirring the resulting mixture at 1500 rpm and at room temperature for 1 h. Then, a “mini-emulsion” was prepared by ultrasonicating the mixture in a S-30-H ultrasonic cleaner (from Elma Schmidbauer GmbH, Singen, Germany) for 5 min. After sonication, the “mini-emulsion” was stirred again at 1500 rpm and at 60 °C for 1h in order to evaporate the chloroform. The P3HT nanoparticles formed due to the insolubility of P3HT in water. The low molecular weight compounds (e.g., SDS) were removed from the solution containing the P3HT nanoparticles by dialysis (using 6–8 kDa molecular weight cut-off, MWCO, membrane from Spectrum Laboratories, Inc., Rancho Dominguez, CA, USA). P3HT-PCBM nanoparticles were synthesized in a similar manner. However, the solution made in chloroform (1 mL) contained not only 13 mg P3HT but also 19.5 mg PCBM.

### 2.3. Preparation of P3HT-Coated Glass Substrates

Thin films of P3HT were deposited onto glass coverslips (22 mm × 22 mm × 0.17 mm, catalog no. 41022012, from Carl Roth GmbH) by spin-coating. The procedure was carried out using solutions of 1% (*w*/*w*) P3HT made in chlorobenzene. Such solutions were stirred for 30 min at 70 °C, cooled down, filtered (using J.T. Baker, 0.2 µm syringe filters made of H-PTFE, from Avantor Inc., Radnor, PA, USA) and stored overnight at 30 °C before being spin-coated onto the glass coverslips. Spin-coating was done at 1000 rpm for 10 s and then at 2000 rpm for 60 s (using a P6700 spin coater from Specialty Coating Systems Inc., Indianapolis, IN, USA). The P3HT-coated glass substrates were annealed at 90 °C for 30 min on a hot plate, thoroughly washed with ultrapure water, and sterilized with 70% ethanol, before being incubated with bEND.3 cells in order to check their biocompatibility.

### 2.4. Characterization of P3HT-Based Materials

Electrochemical characterization was carried out using a single-compartment, three-electrode electrochemical cell in which the working electrode was a glassy carbon electrode (with a 2 mm diameter, disk-shaped electrochemically active area), the reference electrode was a Ag/AgCl, KCl (3 M) electrode, and the counter electrode was a Pt rod. The working electrode was modified with either a P3HT film or P3HT nanoparticles by drop-casting. Photocurrents were recorded with the three electrodes immersed in phosphate-buffered saline (PBS) solution containing 137 mM NaCl, 2.7 mM KCl, 10 mM Na_2_HPO_4_, and 1.8 mM KH_2_PO_4_. They were recorded using an Autolab PGSTAT128N potentiostat (from Metrohm Autolab BV, Utrecht, The Netherlands) while applying 0 mV versus the open circuit potential.

Atomic force microscopy (AFM) imaging was carried out using a NanoWizard II instrument (from JPK Instruments AG, Berlin, Germany) and PPP-NCHR cantilevers (from NanoWorld AG, Neuchâtel, Switzerland). The latter are characterized by a force constant of 42 N/m, a resonance frequency of ~330 kHz, and tip radius of curvature < 10 nm. Images were made using intermittent contact mode in air and relatively low scan rates (e.g., 0.3 Hz), and with the P3HT-based materials deposited onto glass coverslips by drop-casting.

The light absorption properties of the P3HT-based materials were investigated using an Evolution 600 LC UV–Visible spectrophotometer (from Thermo Scientific, Cambridge, UK). Absorbance spectra were corrected by background subtraction in order to remove contributions from the used cuvette and water.

### 2.5. Cells and Their Culturing

Endothelial cells isolated from brain tissue derived from a mouse with endothelioma (bEnd.3, catalog no. CRL-2299, ATCC, Manassas, VA, USA) used as cellular models in the present study were available from Department of Anatomy and Animal Physiology, Faculty of Biology, University of Bucharest. The cells were seeded at a concentration of 2 × 10^5^ cells/mL into 25 cm^2^ cell culture flasks with vent cap (Nunc, EasYFlask NuncIon Delta Surface, Thermo Fischer Scientific Inc., Waltham, MA, USA, cat. no. 156340) and grown until 80% confluence at 37 °C in a 5% CO_2_ humidified incubator (MCO-20AIC Sanyo, Osaka, Japan). The cells were grown in Dulbecco’s modified Eagle’s medium (DMEM), High Glucose with glutamine and sodium pyruvate, supplemented with 10% FBS and penicillin−streptomycin (100 IU/mL–0.1 mg/mL). For subculturing, cells were washed two times with 1 mL PBS and one time with 1 mL trypsin-EDTA 1× solution (diluted from 10× to 1× in PBS). Afterwards, cells were treated with 1 mL trypsin-EDTA 1× solution for 5 min and incubated at 37 °C and 5% CO_2_. The detachment of the cells was confirmed using an inverted transmitted light microscope (Eclipse TS100 from Nikon Corp., Tokyo, Japan). Trypsin-EDTA solution was inactivated with 3 mL of DMEM supplemented with 10% FBS and centrifuged for 5 min at 800 rpm. The resulting pellet was resuspended in 1 mL of fresh culture medium. Cell viability was assessed using the trypan blue exclusion assay and a Neubauer improved counting chamber. All procedures involving cell handling were performed under sterile conditions within a laminar flow biosafety cabinet and all reagents used sterilized by filtration (cellulose filters with 0.22 μm pore diameter), or autoclaved.

### 2.6. Investigation of the Biocompatibility of P3HT Nanoparticles and Films

To assess the biocompatibility of P3HT films, bEnd.3 cells were harvested from culture flasks through trypsinization and seeded onto P3HT-coated glass coverslips, which had been placed on the base of 3.5 cm diameter polystyrene Petri dishes (catalog no. 627161, from Greiner Bio-One International GmbH, Kremsmünster, Austria). After 48 h of incubation, the medium bathing the P3HT-coated coverslips was replaced with fresh one. Subsequently, the cells growing on the P3HT-coated coverslips were exposed to three series of light pulses over a 24 h period. The light pulses were produced with a warm white Luxeon Rebel LED, mounted on a 20 mm Star CoolBase (from Luxeon Star LEDs, Lethbridge, AB, Canada). They were 1 s long, spaced 1 s apart, and characterized by an LPD = 1.3 mW/cm^2^. The three series consisted of 900 pulses each (thus, totaling 2700 pulses) and were spaced approximately 8 h apart. For comparison purposes, cells cultured on uncoated coverslips were also investigated. These control experiments included both cells subjected to the above-described light pulses and cells that were not subjected to light pulses. To assess the biocompatibility of P3HT-based nanoparticles, bEnd.3 cells were harvested from culture flasks through trypsinization and seeded onto 3.5 cm diameter glass bottom Petri dishes (FluoroDishTM, from World Precision Instruments, Sarasota, FL, USA). After 4 h, the cells which did not properly adhere to the base of the Petri dishes were removed by washing with culture medium and the remaining cells were incubated with either P3HT nanoparticles or P3HT-PCBM nanoparticles suspended in culture medium. The nanoparticle concentration, the cells were exposed to, was standardized to achieve an optical absorption of 0.5 at λ ~ 570 nm and L = 1 cm (where L is the light path length). This optical absorption corresponds to an estimated nanoparticle concentration of 7.6 µg/mL (see also Section S5 of the Supplementary Materials), which lies well within the range commonly employed in nanoparticle cytotoxicity studies (1–100 µg/mL). After 48 h of incubation, the cell culture medium bathing the bEnd.3 cells was replaced with fresh medium (during which most of the nanoparticles which did not enter into the cells or did not adhere to the cells were also removed). Subsequently, the cells carrying P3HT-based nanoparticles (within their cytoplasm and on their surfaces) were subjected to the above-described light exposure program. For comparison purposes, control cells cultured on glass-bottom Petri dishes were also investigated under an identical protocol. These control experiments included cells exposed to light pulses and cells that were not exposed to light pulses. The biocompatibility tests carried out within the present study are summarized in Table 1. The same table introduces notations for the different experimental groups.

### 2.7. MTT Assay

Metabolically active cells use NAD(P)H-dependent oxidoreductase enzymes to reduce the yellow tetrazolium salt MTT to insoluble purple formazan crystals. This particularity of metabolically active cells is exploited in the MTT assay. To carry out the assay, thiazolyl blue was added to the Petri dish containing either treated or untreated (i.e., reference) cells to a final concentration of 0.5 mg/mL and incubated for 4 h to allow viable cells to convert the reagent into purple formazan. The MTT solution was then removed and the insoluble purple formazan crystals generated by the viable cells were dissolved using DMSO. The absorbance of the resulting purple colored solution was next measured using a microplate reader (Tecan Infinite 200 Pro, Tecan Trading AG, Männedorf, Switzerland) at λ = 620 nm. The amount of light absorbed by the solution recovered from the untreated cells (i.e., -P3HT film/-light or -P3HT NPs/-light) was considered to correspond to 100% of viability. Biocompatibility tests were performed with three independently prepared batches of P3HT-based materials, tested with bEND.3 cells from three different passages/seedings; for each passage, cells were exposed in triplicate wells, yielding n = 9 per condition. Changes in viability were evaluated for statistical significance using one way ANOVA and three different significance levels (0.05, 0.01, and 0.001).

## 3. Results and Discussion

Before investigating their effect on the viability of endothelial cells, the P3HT-based materials were characterized using AFM, spectrophotometry and photoelectrochemistry (see Section 3.1). The aim was to show that the materials used within the present study reproduce the main features reported in the literature for such materials (e.g., light absorption properties, photocurrent generation, etc.). bEND.3 cells were subsequently incubated with the three, P3HT-based materials (see Section 3.2). Some of the cells were also exposed to a photoexcitation characterized by LPD we normally encounter in office areas and outdoor on a moderately sunny day (i.e., 1.3 mW/cm^2^). This LPD was selected because a very promising use of P3HT-based materials is to replace faulty photoreceptors in eyes affected by macular degeneration (as shown in Refs. [17,19,42]).

### 3.1. Properties of the Investigated P3HT-Based Materials

**Figure 1:**
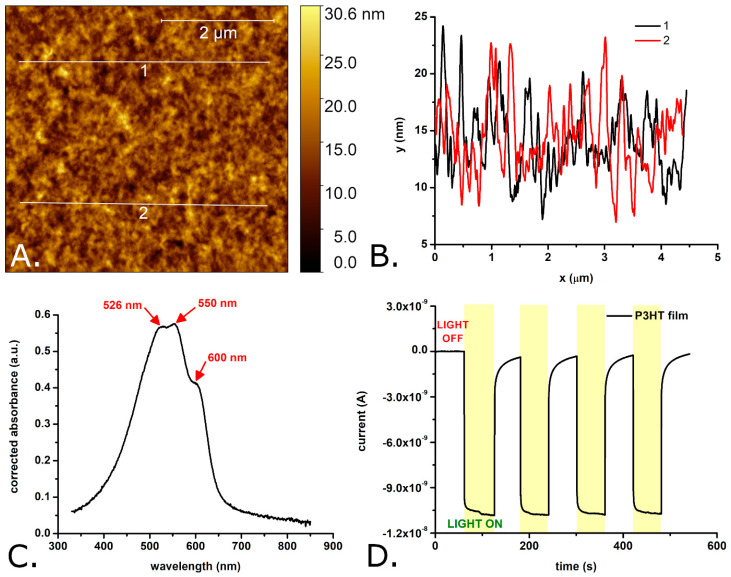
AFM image of a P3HT film spin coated onto a glass substrate (**A**), profiles extracted from the AFM image showing the rugosity of the film (**B**), absorption spectrum of P3HT-coated glass (**C**), and photocurrents obtained with glassy carbon electrodes modified with a P3HT film (**D**). Observations: The glassy carbon electrode modified with the P3HT film was periodically illuminated with a white light LED placed 2 cm from the modified electrode surface. The LPD at the P3HT film was 1.3 mW/cm^2^.

Figure 1 provides an overview of the main properties of the P3HT films which were presented to the bEND.3 cells in the present study.

As shown in Figure 1A,B, the surface of the investigated P3HT films is relatively smooth, with a rugosity in the nanometers range. The difference in between the maximum peak height and the maximum pit depth of the P3HT films was measured to be 28.2 ± 6.6 nm, the mean roughness (R_a_) was determined to be 2.4 ± 0.7 nm, while the RMS roughness (R_q_) was determined to be 3.0 ± 0.8 nm. This RMS roughness is slightly higher than that of plain glass coverslips (e.g., 1.2 ± 0.6 nm in [43]) and of P3HT films spin coated onto silicon wafers (e.g., 0.4 ± 0.1 nm in [5]) but lower than the roughness reported by some authors for P3HT films spin coated onto glass substrates (e.g., 5.5 nm in [29]). However, previous studies [44] indicate that these roughness values do not differ enough to cause roughness-based variations in the growth of bEnd.3 cells on P3HT films compared to those grown on glass coverslips. The average thickness of the P3HT films was 82.4 ± 2.2 nm (see also Figure S1 of the Supplementary Materials). The absorption spectrum of the P3HT films (see Figure 1C) shows three, partially overlapping absorption peaks with their maxima positioned at 526 ± 1 nm, 550 ± 1 nm, and 600 ± 1 nm. These absorption peaks are due to the π–π* transitions induced by the green–orange region of the visible spectrum in the conjugated π bonds system of P3HT. The positions of the absorption peaks match very well the positions of the absorption peaks of regioregular P3HT films described in the literature (e.g., 523 nm, 546 nm and 593 nm in Ref. [45]). Typical currents generated by the P3HT films under the effect of light are shown in Figure 1D. Important to note, these currents were measured with P3HT films immersed into aqueous solution and while applying 0 mV versus the open circuit potential, conditions which match well those encountered by P3HT in biological systems (i.e., aqueous microenvironment and no easy possibility to set the polymer to a certain potential). As one can observe, the photocurrents are characterized by an initial fast rise followed by a rather slow rise and (once the light is turned off) by an initial fast decay followed by a long-lived tail. The photocurrents reach 90% of their maximum in roughly 0.5 s. Such a slow turn on dynamics is considered a sign of poor charge transport properties [46,47]. This could be due to the thicker films which were deposited by drop casting onto the electrodes (compared to the films deposited by spin coating onto the glass substrates). However, the P3HT films are clearly photoactive and able to generate photoelectrons and, thus, photocurrents which do not show significant amplitude variations from one light pulse to another. According to previous studies (e.g., [23,37]), the electrons generated by the P3HT material under the effect of light end up being transferred to the oxygen dissolved in the solution bathing the P3HT material. In turn, the reduction of the oxygen dissolved in the solution leads to ROS (such as superoxide ions and hydrogen peroxide).

**Figure 2:**
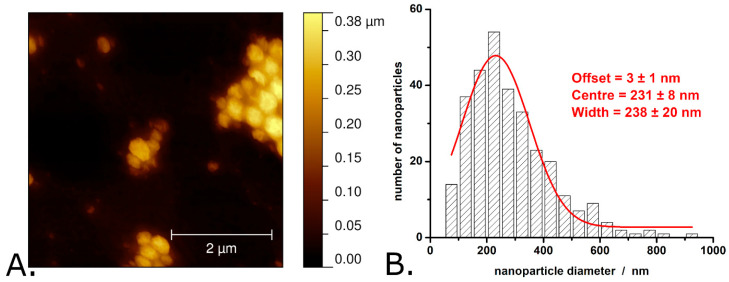
AFM image of P3HT nanoparticles (**A**) and the distribution of P3HT nanoparticle diameters as determined from AFM images (**B**). Observations: The AFM image shown here was obtained in air, with the nanoparticles deposited onto a glass slide. The nanoparticle aggregates shown in the image formed during the evaporation of water from the nanoparticle suspension drop deposited on the glass slide. The nanoparticle suspension itself is stable, without pronounced signs of aggregation or sedimentation.

**Figure 3:**
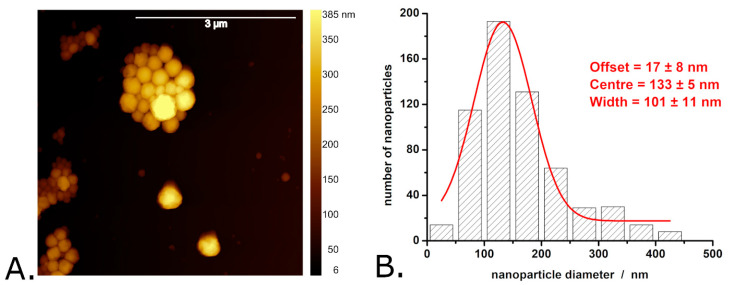
AFM image of P3HT-PCBM nanoparticles (**A**) and the distribution of P3HT-PCBM nanoparticle diameters determined from AFM images (**B**). Observations: The AFM image shown here was obtained in air, with the nanoparticles deposited onto a glass slide. The aggregates visible in the image formed during the evaporation of water from the nanoparticle suspension drop deposited on the glass slide. The nanoparticle suspension itself is stable, without pronounced signs of aggregation or sedimentation.

**Figure 4:**
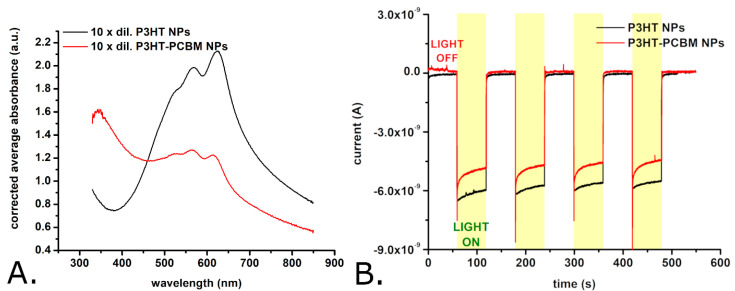
Absorption spectra of suspensions of P3HT nanoparticles and of P3HT-PCBM nanoparticles (**A**), and photocurrents obtained with glassy carbon electrodes modified with either P3HT nanoparticles or P3HT-PCBM nanoparticles (**B**). Observations: The nanoparticle suspensions were diluted by a factor of 10 for recording the absorption spectra shown here. The initial nanoparticle suspension contains approximately 13 mg/mL of nanoparticles. The glassy carbon electrode modified with P3HT nanoparticles was periodically illuminated with a white light LED placed 2 cm from the modified electrode surface. The LPD at the electrode level was 1.3 mW/cm^2^.

Figure 2, Figure 3 and Figure 4 present typical results obtained during the characterization of P3HT and P3HT-PCBM nanoparticles produced via the “mini-emulsion” method described in the Section 2. AFM was used to determine the shape and diameter of the P3HT nanoparticles (see Figure 2A,B) and of the P3HT-PCBM nanoparticles (see Figure 3A,B) (additional AFM images of the nanoparticles are shown in Figure S2 of the Supplementary Materials). Analysis of the AFM images revealed that the P3HT nanoparticles have a quasi-spherical shape (see Figure 2A).

The average diameter of P3HT nanoparticles is 280 ± 150 nm. The exact distribution of P3HT nanoparticle diameters is shown in Figure 2B and indicates that the P3HT particle diameters exhibit significant dispersion (with the width of the Gaussian fit being as large as 240 nm). Most of the P3HT nanoparticles (i.e., approximately 30%) have diameters between 175 and 275 nm, while ~2% of the P3HT nanoparticles have diameters greater than 625 nm. In terms of size and size distribution, these P3HT nanoparticles resemble very much the P3HT nanoparticles made by similar procedures in other studies (see, for example, Refs. [19,37]). AFM images of the P3HT-PCBM nanoparticles revealed that these nanoparticles also have a quasi-spherical shape (see Figure 3A).

However, the surface of the P3HT-PCBM nanoparticles is somewhat smoother than that of the P3HT nanoparticles. The P3HT-PCBM nanoparticles feature an average diameter of 169 ± 99 nm (and thus are somewhat smaller than the synthesized P3HT nanoparticles). The exact distribution of P3HT-PCBM nanoparticle diameters is shown in Figure 3B, and indicates that the P3HT-PCBM particle diameters exhibit a smaller dispersion than the above described P3HT particle diameters (the width of the Gaussian fit being ~100 nm vs. ~240 nm). Most of the obtained P3HT-PCBM particles (i.e., approximately 30%) have diameters between 100 and 150 nm, while only ~2% of the P3HT-PCBM nanoparticles have diameters greater than 450 nm. Regarding the ability of the synthesized nanoparticles to absorb light, we found that the absorption spectra of the P3HT nanoparticles and of the P3HT-PCBM nanoparticles (just as the absorption spectra of the P3HT films described above) show absorption peaks characteristic of the π–π* transitions of regioregular P3HT [45,48,49] (see Figure 4A).

These absorption peaks have maxima at 532 ± 2 nm, 568 ± 1 nm, and 623 ± 1 nm in case of the P3HT nanoparticles and 527 ± 2 nm, 565 ± 1 nm, and 614 ± 1 nm in the case of P3HT-PCBM nanoparticles. In yet other words, the spectra of the P3HT nanoparticles and of the P3HT-PCBM nanoparticles are slightly redshifted with respect to the spectra of the P3HT films. Such a redshift is usually attributed to an increase in the conjugation lengths of the polymer chain segments. The relative magnitudes of the absorption peaks (found at λ ~ 600 nm and at λ ~ 550 nm) indicate that the nanoparticles (and first of all the nanoparticles made without PCBM) contain much more J-aggregates than the P3HT films which are dominated by H-aggregates [50]. While the absorption spectra of the P3HT films (see Figure 1C) and of the P3HT nanoparticles (see Figure 4A) show no significant absorption at wavelengths below 400 nm, the absorption spectrum of the P3HT-PCBM nanoparticles shows important absorption also in between 300 nm and 400 nm (see Figure 4A). This absorption is due to the PCBM found within the P3HT-PCBM nanoparticles [49,51]. Just as it was done for the P3HT films, the AFM and spectrophotometric investigations were complemented with the evaluation of the photocurrents generated by the P3HT nanoparticles and the P3HT-PCBM nanoparticles. Figure 4B shows the photocurrents generated by electrodes modified with the two P3HT-based nanoparticles. As one can observe, the photocurrents generated by the two P3HT-based nanoparticles are comparable in amplitude. Interestingly enough, the photocurrents of the nanoparticles have a much faster turn-on and turn-off dynamics than the photocurrents generated by the P3HT films (see Figure 1D versus Figure 4B). As the particles and the films were made using the same polymer batch, the slower dynamics of the photocurrents generated by the films are most probably due to the significant thickness of the films. (Observation: The P3HT films incubated with bEND.3 cells, see Section 3.2, were obtained by spin coating glass coverslips with P3HT. These films had a thickness of only 82.4 ± 2.2 nm as determined with AFM. However, due to the lack of electrical conductivity of the glass substrate, these films were not suitable for photoelectrochemical measurements. Consequently, the photoelectrochemical properties of the P3HT films were studied using thicker films created by drop casting P3HT onto glassy carbon electrodes.)

### 3.2. Viability of bEND.3 Cells in Contact with Different Forms of P3HT, with and Without Illumination

As a first step in the investigation of the biocompatibility of P3HT-based materials, the compatibility of P3HT films with bEND.3 cells was investigated. bEND.3 cells provide versatile in vitro models for blood–brain barrier endothelia. The cells, murine immortalized brain endothelial cell line, have typical endothelial morphology and adherent growth properties. To this end, cells were grown onto either bare glass coverslips or P3HT-coated glass coverslips. After 48 h, some of the coverslips carrying the cells were subjected to trains of light pulses while others were kept in dark (see the exact details of the light stimulation in the Section 2 and in Figure S3 of the Supplementary Materials). Once the photoexcitation protocol was finished, the proliferation and the viability of the cells was evaluated using the MTT assay.

**Figure 5:**
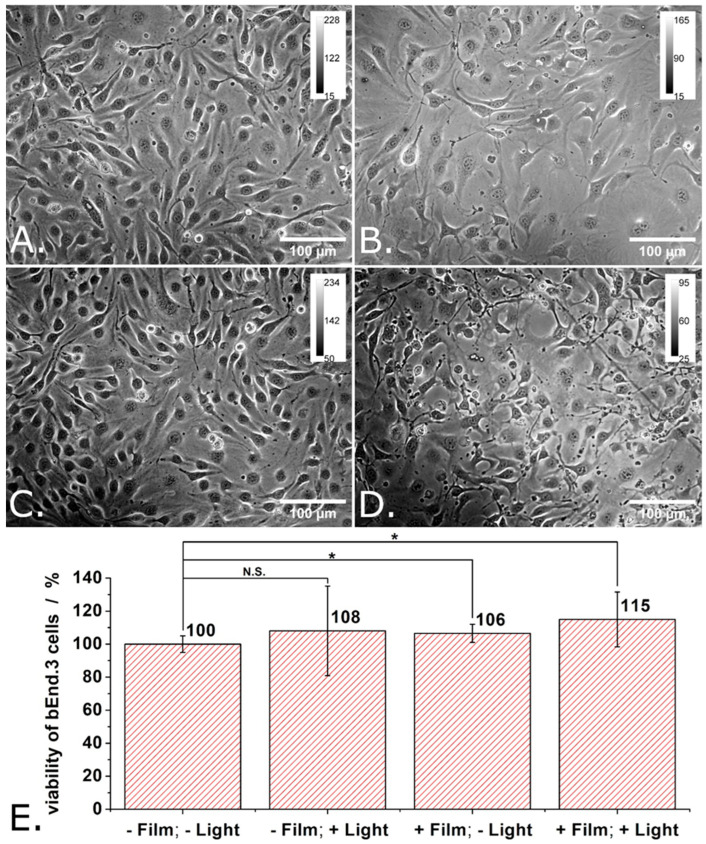
Transmitted light microscopy images of “reference” bEND cells (**A**), of bEND cells grown onto P3HT-coated glass (**B**), of reference bEND cells subjected to light pulses (**C**), and of bEND cells grown onto P3HT-coated glass and subjected to light pulses (**D**), and the viability of such cells (**E**). Experimental conditions: Cells were grown onto either glass slides or P3HT-coated glass slides for 48 h. After 48 h, the culture medium was renewed and the cells were further cultivated for another 24 h. For the illumination protocol, three trains of light pulses (at LPD = 1.3 mW/cm^2^) distributed over 24 h were applied. Each train contains 900 pulses of 1 s duration and 0.5 Hz repetition rate. After 24 h the MTT assay was performed. Asterisks indicate levels of statistical significance: *p* < 0.05 (*).

Figure 5A–D show transmitted light microscopy images of the cells grown in the 4 different experimental conditions (i.e., -P3HT film/-light, -P3HT film/+light, +P3HT film/-light, and +P3HT film/+light).

When grown for 48 h onto glass, the bEND.3 cells have grown to full confluence, adopted elongated shapes (characteristic for endothelia) and showed an important tendency to align with each other (see Figure 5A). Meanwhile, when grown for 48 h onto P3HT-coated glass, the bEND.3 cells have grown to medium densities, adopted polygonal shapes quite often, and showed higher occurrence of enlarged morphologies and only a limited tendency to align with each other (see Figure 5B). Cell proliferation rate appears lower on P3HT-coated glass compared to glass. As one can observe in Figure 5C,D, while the differences in cell density fade in time, the differences in cell shape tend to persist and cells remain elongated when grown onto glass and polygonal when grown onto P3HT.

Figure 5E shows the proliferation and the viability of the bEND.3 cells grown in different conditions as evaluated using the MTT assay and considering the cells grown onto glass and kept into dark (i.e., -P3HT film/-light) as being the 100% viability control. Just as expected (based on the transmitted light microscopy images made during these experiments), there are only small differences in the viability of the cells grown in the 4 different experimental conditions in the sense that both the P3HT film and the photoexcitation protocol increase a little bit the proliferation and the viability of bEND.3 cells at 96 h after seeding (see Figure 5E). Compared to this, significant viability increases (e.g., 158%) were previously observed for ECFCs grown on illuminated P3HT films as compared to ECFCs grown on glass, on illuminated glass or on P3HT films kept in dark [6]. The viability increase was attributed by the authors of the respective study to the optical modulation of the Ca^2+^-permeable TRPV1 channel. A follow up study indicated that this channel is “gated” by the hydrogen peroxide produced by the photoexcited P3HT film [7]. Other authors reported a ~ 45% decrease in the viability for HEK-293 cells stably transfected with the human TRPV1 channel and grown onto P3HT films [15]. Interestingly enough, they observed such a decrease in the viability of the cells although their P3HT films were coated with fibronectin before being presented to the cells. The authors of the respective study also bring arguments for the activation of the human TRPV1 channels by local temperature increases and local pH variations rather than hydrogen peroxide [15]. Our results combined with those of previous studies indicate that the cytotoxicity of P3HT films must be carefully investigated as it depends on multiple details of the experimental system.

**Figure 6:**
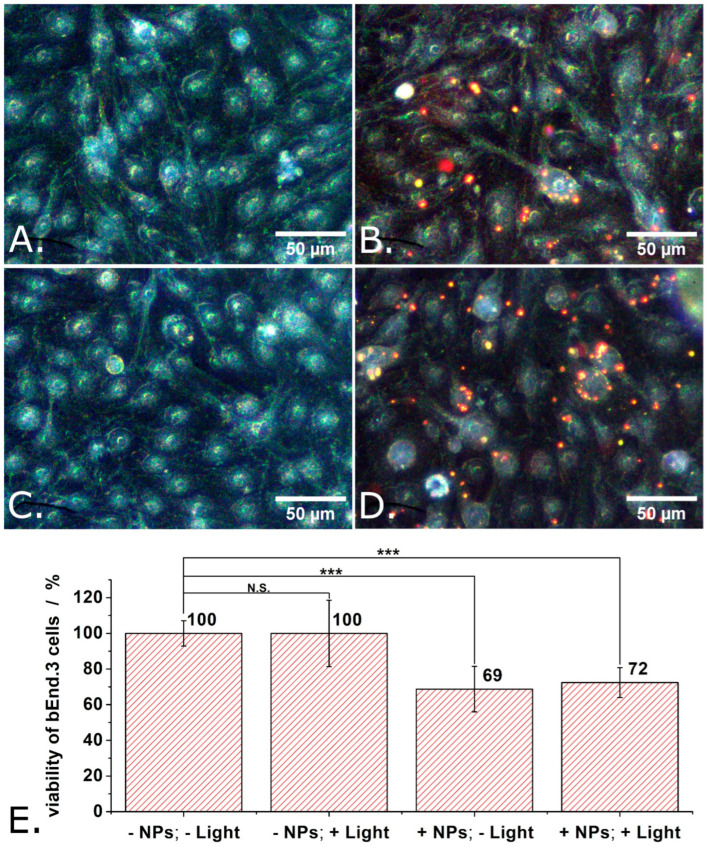
Dark field microscopy images of control bEND cells, on glass support (**A**), of bEND cells incubated with P3HT nanoparticles (**B**), of control bEND cells subjected to light pulses (**C**), and of bEND cells incubated with P3HT nanoparticles and subjected to light pulses (**D**), and the viability under these conditions (**E**). Experimental conditions: Treated cells were incubated for 48 h with P3HT nanoparticles. After 48 h, the culture medium of cells was changed and the cells remained in contact, for another 24 h, with only the P3HT nanoparticles which were internalized or adsorbed on their membrane. During those 24 h, the cells were subjected to a train of 1 s long light pulses, distanced at 1 s from each other, for 30 min. This train of light pulses (at LPD = 1.3 mW/cm^2^) was repeated 3 times over 24 h, after which the MTT assay was performed. Asterisks indicate levels of statistical significance: *p* < 0.001 (***).

In the next step of the investigation, the compatibility of the P3HT nanoparticles with bEND.3 cells was determined. Due to the particulate nature of the investigated material, the experimental protocol used with the P3HT films was slightly modified. The P3HT films were presented to the bEND.3 cells as substrate to adhere and grow onto; in contrast, the P3HT nanoparticles were added into the extracellular space of bEND.3 cells 4 h after seeding onto glass bottom Petri dishes. From this point on, the experiments with the P3HT nanoparticles were carried out just as those with the P3HT films. The medium of the cells was changed with fresh one at 48 h after cell seeding (obs.: this removed most P3HT nanoparticles which did not enter the cells or adhere well to cells). Then, the train of light pulses described above was used to photoexcite the P3HT nanoparticles. The photoexcitation protocol was applied 3 times in 24 h. Finally, the viability of the cells was evaluated using the MTT assay (see also Figure S3 of the Supplementary Materials). Figure 6 shows not only the results of the MTT assays carried out on the cells incubated with P3HT nanoparticles but also representative dark field microscopy images of the individual experimental conditions. Switching from transmitted light microscopy used above (see Figure 5) to dark field microscopy was motivated by the good visibility of the P3HT nanoparticles in the latter (due to light scattering). As one can observe in Figure 6B,D, certain bEND.3 cells were in contact with several P3HT nanoparticles while other bEND.3 cells were free of P3HT nanoparticles. In yet other words, the bEND.3 cells were clearly not exposed to an exaggerated number of P3HT nanoparticles. In spite of this fact, the proliferation of adherently growing bEND.3 cells decreased by about 30% when incubated with P3HT nanoparticles (see Figure 6E). Observation: Means of 9 independent experiments were compared using one way ANOVA and the means of cells treated with P3HT nanoparticles were found to be significantly different from the means of untreated cells at a significance level of 0.001. This outcome is to some extent unexpected if one considers our results obtained with P3HT films and some previous findings described in the literature. For instance, P3HT nanoparticles with diameters significantly smaller than ours (i.e., 60 nm vs. 280 nm) were reported to not impact the proliferation of HUVECs at all [22].

Moreover, when they were made porous, P3HT nanoparticles were still harmless for HUVECs [22]. P3HT nanoparticles (aggregated into assemblies with diameters around 0.5 µm) were neither endocytosed nor harmful to retinal cells [19,20]. However, P3HT nanoparticles (with average diameters ranging from 150 nm to 240 nm) were found to reduce the viability of HEK-293 cells with 20% to 40% (depending also on the length of incubation) [36,37]. As also shown in Figure 6E, light does not seem to add to the cytotoxicity of our P3HT nanoparticles. This could be due to the low LPD used in our experiment (only 1.3 mW/cm^2^, corresponding to LPD we normally encounter in office areas and outdoor on a moderately sunny day). A comparable (but still somewhat larger LPD, i.e., 6 mW/cm^2^) was previously observed to not alter in any way the cytotoxicity of P3HT nanoparticles when this was tested with HUVECs [22] or with primary cortical neurons from rats [10]. However, P3HT nanoparticles excited with light at LPD of 9500 mW/cm^2^ did show cytotoxicity when this was applied on HEK-293 cells [37]. Studying the effect of such high LPDs was not among our aims, as our focus is on exploring the potential use of P3HT nanoparticles in interventions on diseased retinas. Typically, retinas are not exposed to such high LPDs.

**Figure 7:**
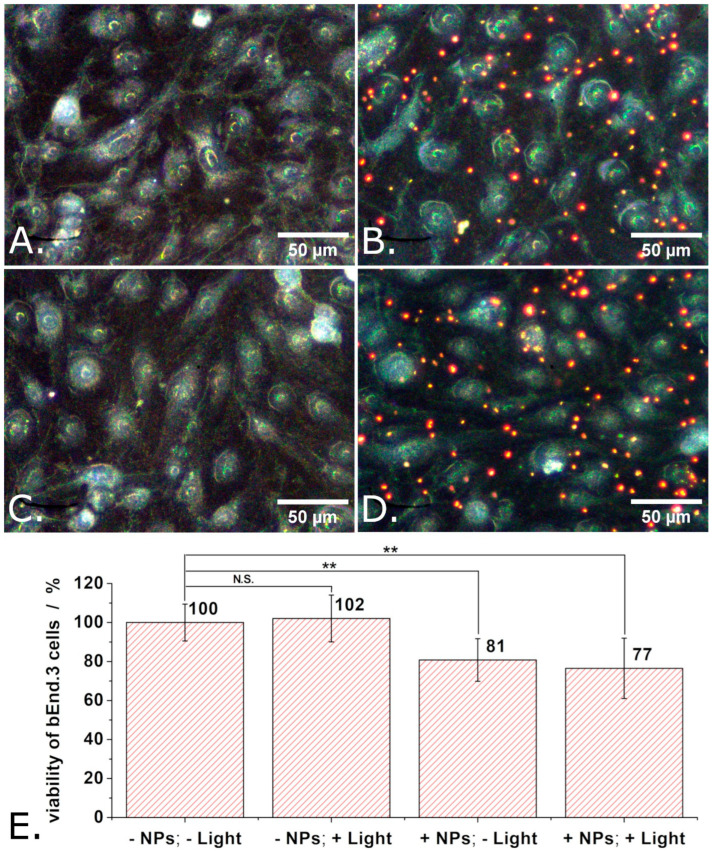
Dark field microscopy images of “reference” bEND cells (**A**), of bEND cells incubated with P3HT-PCBM nanoparticles (**B**), of bEND cells subjected to light pulses (**C**), and of bEND cells incubated with P3HT-PCBM nanoparticles and subjected to light pulses (**D**), and the viability of such cells (**E**). Experimental conditions: Treated cells were incubated for 48 h with P3HT-PCBM nanoparticles. After 48 h, the culture medium of such cells was changed and the cells remained in contact, for another 24 h, with only the P3HT-PCBM nanoparticles which were internalized or adsorbed on their membrane. During those 24 h, the cells were subjected to a train of 1 s long light pulses, distanced at 1 s from each other, for 30 min. This train of light pulses (at LPD = 1.3 mW/cm^2^) was repeated 3 times over 24 h, after which the MTT assay was performed. Asterisks indicate levels of statistical significance: *p* < 0.01 (**).

Similar experiments but in which the P3HT nanoparticles were replaced with P3HT-PCBM nanoparticles were also carried out. The obtained results are summarized in Figure 7. As summarized in Figure 7E, the proliferation of adherently growing bEND.3 cells decreased by about 20% when incubated with P3HT-PCBM nanoparticles. Observation: Means of 9 independent experiments were compared using one way ANOVA and the means of cells treated with P3HT-PCBM nanoparticles were found to be significantly different from the means of untreated cells at a significance level of 0.01. This decrease in the viability of the bEnd.3 cells is somewhat smaller than that induced by the P3HT nanoparticles (20% vs. 30%) and still not significantly affected by illumination.

The results shown in Figure 6E and Figure 7E taken together and compared to the results shown in Figure 5E indicate that the decreased viability of the cells incubated with P3HT-based nanoparticles has to do with the P3HT being presented to the cells as nanoparticles which interact not only with the surface of the cell but also with its intracellular organelles (see also Figure S4 of the Supplementary Materials). Somewhat unexpectedly (taking into account that they could lead to the production of ROS [23]), the photoelectrons generated by the P3HT nanoparticles under the effect of the illumination used in the present study seem not to add to the cytotoxicity of the P3HT nanoparticles. Noteworthy, while the cytotoxicity of nanoparticles is most often mediated by the generation of ROS [52], cytotoxicity independent of oxidative stress has also been reported [53]. Moreover, the physical form of nanoparticle (including size, shape, surface roughness, and surface chemistry) can substantially affect the extent of cellular uptake, intracellular localization, and resulting biological impact as highlighted in several reviews [54,55,56]. The mechanistic basis of the form-dependent cytotoxicity observed here remains to be clarified. Among the factors most likely to contribute are (i.) the differential uptake of P3HT nanoparticles compared to P3HT films, and (ii.) altered cell–P3HT surface interactions. Internalization of P3HT nanoparticles may trigger cellular responses such as oxidative stress, while altered surface interactions may compromise cell membrane integrity. Such mechanisms have been widely discussed in the nanotoxicology literature (e.g., in Refs. [57,58,59,60]), and further studies will be needed to clarify which of them underlies the effects observed here.

## 4. Conclusions

One promising use of P3HT-based materials is in the replacement of damaged photoreceptors of the retina (see Refs. [17,19,42]). The retina is a vascularized tissue, which means that endothelial cells are present within its structure. Thus, when used in the retina, P3HT must be compatible both with the different cell types directly involved in vision (e.g., bipolar cells, ganglion cells, etc.) and with endothelial cells which line all blood vessels. Motivated by these facts, the compatibility of P3HT films and P3HT nanoparticles with mouse endothelial cells (specifically with bEnd.3 cells) was comparatively investigated for the first time. The said compatibility was investigated both in the presence and in the absence of a photoexcitation protocol consisting of trains of relatively long light pulses (i.e., 1 s) at LPD = 1.3 mW/cm^2^, comparable with the LPD we normally encounter in office areas and outdoor on a moderately sunny day.

It was discovered that, while P3HT films have no detrimental effect on the viability of bEnd.3 cells, the P3HT nanoparticles decrease the viability of bEnd.3 cells by about 30% when they contain no PCBM and by about 20% when they contain PCBM. The form in which P3HT is presented to living cells clearly plays a very important role in the biocompatibility of this material (as is true for other materials as well [38]). Although the reductions in cell viability induced by the P3HT-based nanoparticles are moderate in magnitude, they are reproducible and statistically robust. Such partial decreases in viability can be biologically meaningful depending on the context: for example, even modest losses of endothelial cells may impair vascular integrity if sustained in vivo, an effect that may be tolerated in some tissues but could have pronounced consequences in sensitive organs (such as the retina or the brain). To determine whether the observed viability loss translates into impaired endothelial function, additional investigations are needed. These should clarify whether the effect is transient, concentration-dependent, or likely to have pathological consequences. Interestingly enough, “mild” photoexcitation of the P3HT-based materials did not significantly affect their compatibility with the bEnd.3 cells. These results do not exclude a potential role of ROS in the observed cytotoxicity; rather, they indicate that, the observed negative impact of P3HT nanoparticles and of P3HT-PCBM nanoparticles on the viability of the bEnd.3 cells is not substantially mediated by the photochemical (e.g., direct ROS generation) and photophysical processes produced by such nanoparticles.

Several important questions remain open. Future work should therefore focus on (i). comparative studies with P3HT nanoparticles of different sizes to determine whether cytotoxicity is linked to particle diameter, (ii). investigating how nanoparticle surface chemistry influences biocompatibility, (iii). extending experiments to multiple cell types to assess broader biological relevance, (iv). structural characterization of nanoparticles (e.g., crystallinity, strain, phase composition, etc.) to relate material properties to cellular responses, (v). elucidating the mechanisms underlying cytotoxicity (e.g., oxidative stress, membrane disruption, etc.), and (vi). examining the dynamics of nanoparticle localization at the cellular level.

## Figures and Tables

**Table 1:** Experimental groups (combining bEnd.3 cells, P3HT-based materials, and light stimulation) investigated within the present study. Observation: Experimental groups no. 1, 5, and 9 are all negative control groups. However, they were investigated separately and in parallel with the corresponding P3HT-treated groups. Experimental groups 2, 6, and 10 are also equivalent. However, they were also investigated separately and in parallel with the corresponding P3HT-treated groups.

No.	P3HT-Based Material	Light Stimulation	Notation for the Experimental Group
1	no	no	-P3HT film/-light
2	no	yes	-P3HT film/+light
3	P3HT film	no	+P3HT film/-light
4	P3HT film	yes	+P3HT film/+light
5	no	no	-P3HT NPs/-light
6	no	yes	-P3HT NPs/+light
7	P3HT nanoparticles	no	+P3HT NPs/-light
8	P3HT nanoparticles	yes	+P3HT NPs/+light
9	no	no	-P3HT-PCBM NPs/-light
10	no	yes	-P3HT-PCBM NPs/+light
11	P3HT-PCBM nanoparticles	no	+P3HT-PCBM NPs/-light
12	P3HT-PCBM nanoparticles	yes	+P3HT-PCBM NPs/+light

## Data Availability

The original contributions presented in this study are included in the article/Supplementary Materials. Further inquiries can be directed to the corresponding author.

## Supplementary Materials

The following supporting information can be downloaded at: https://www.mdpi.com/article/10.3390/ma18204671/s1, PDF file with the biocompatibility of P3HT films and P3HT nanoparticles as reported in the literature (Table S1), and with 4 figures related to the thickness of the P3HT films presented to the bEnd.3 cells (Figure S1), the shapes of the P3HT nanoparticles and of the P3HT-PCBM nanoparticles as revealed with AFM (Figure S2), the timeline of the biocompatibility tests conducted on the P3HT films and P3HT nanoparticles (Figure S3), and the localization of the P3HT-based nanoparticles at cellular level (Figure S4). The same PDF file describes the estimation of the concentration of P3HT-based nanoparticles from absorbance data (Section S5), and lists all the viability values measured in the different experimental conditions of our study (Tables S2–S4).
