# Supplementary material for: The Impact of Physical Form on the Biocompatibility of Poly(3-hexylthiophene-2,5-diyl)
Source: Materials (Basel). 2025 Oct 11;18(20):4671. doi: 10.3390/ma18204671 (PMC12565759; doi:10.3390/ma18204671)
Supplement: Supplementary file 1 [file materials-18-04671-s001.zip › materials-3893313-supplementary.pdf]

Supplementary Material for:

The impact of physical form on the biocompatibility of poly(3-hexylthiophene-2,5-diyl)

Daniela A. Tudor[1,2], Sorin David[1], Mihaela Gheorghiu[1,2], Szilveszter Gáspár[1*]

[1] International Centre of Biodynamics, 1B Intrarea Portocalelor, 060101 – Bucharest, Romania
[2]Faculty of Biology, University of Bucharest, 91-95 Splaiul Independenței, 050095 – Bucharest, Romania
** Corresponding author; sgaspar@biodyn.ro*

Contents

S1. Reported in vitro biocompatibility outcomes of P3HT films and P3HT nanoparticles across different cell models and illumination conditions 2

S2. Thickness of the poly(3-hexylthiophene-2,5-diyl) (P3HT) films presented to bEnd.3 cells 4

S3. AFM images of the P3HT-based nanoparticles presented to the bEnd.3 cells 4

S4. Schematic representation of the timeline of the biocompatibility tests 5

S5. Estimating the concentration of nanoparticles from absorbance data 6

S6. Estimating the localization of P3HT-based nanoparticles at cellular level from dark-field microscopy images 6

S7. Viability values measured while testing the compatibility of the P3HT-based materials with bEnd.3 cells 7

S8. References 9

S1. Reported in vitro biocompatibility outcomes of P3HT films and P3HT nanoparticles across different cell models and illumination conditions

Table S1 summarizes significant part of the scientific literature on the biocompatibility of P3HT films and P3HT nanoparticles, as assessed using different cellular models and illumination conditions. Overall, the data indicate that P3HT films and P3HT nanoparticles have been reported as both biocompatible and cytotoxic, depending on the specific experimental context.

Table S1. *Reported in vitro biocompatibility outcomes of P3HT films and P3HT nanoparticles across different cell models and illumination conditions. (Observation: → indicates no effect, ↓ indicates detrimental effect, and ↑ indicates improvement as compared to reference cells grown in the absence of the P3HT-based material.)*

P3HT form	Size / Structure	Cellular model	Illumination parameters	Reported effect (assay)	Ref.
Film	150 nm thick film that underwent oxygen plasma treatment	Human adipose-derived stem cells	Dark	→ Proliferation (Alamar blue assay)	[4]
	No further details provided	Human adipose-derived mesenchymal stem cells	Dark	↑ Proliferation (MTT assay)	[5]
	150 nm thick film	Endothelial colony-forming cells	525 nm, 40 mW/cm²	↑ Proliferation (counting)	[6]
	P3HT film was coated with poly-L-lysine	Primary rat embryo hippocampal neurons	Dark	→ Viability (fluorescein diacetate and 4’,6-diamidino-2-phenylindole staining)	[13]
	~ 250 nm thick film coated with fibronectin	Human embryonic kidney 293 cells	Dark	↓ Proliferation (MTT assay)	[15]
	200 – 250 nm porous P3HT film coated with fibronectin	Human umbilical vein endothelial cells	520 nm, 110 mW/cm^2	→ Proliferation (Alamar blue assay)	[21]
	P3HT mixed (or not) with carbon nanotubes	Mouse Hippocampal Neuronal Cell Line (HT-22)	Dark	↑ Viability (MTT assay)	[28]
	100 nm thick P3HT film coated with fibronectin	Human embryonic kidney 293 cells	Dark	→ Proliferation (MTT assay)	[30]

P3HT form	Size / Structure	Cellular model	Illumination parameters	Reported effect (assay)	Ref.
	P3HT:PCBM film coated with poly-d-lysine	Primary rat neocortical astrocytes	Dark	→ Viability (fluorescein diacetate staining)	[32]
	100 nm – 10 µm thick film	L929 mouse fibroblasts	Dark	↓ Viability (optical microscopy)	[33]
	30 – 50 nm thick film	Mouse 3T3 fibroblasts	Dark	↓ Viability (MTT assay)	[34]
	80 nm thick film	Mouse brain capillary endothelial cells (bEND.3)	White light, 1.3 mW/cm^2	↑ Viability (MTT assay)	This work
Nanoparticle	~ 70 - 100 nm	Bone marrow mesenchymal stem cells	Dark	→ Proliferation (MTT assay)	[10]
	~ 70 - 100 nm, entrapped into a collagen hydrogel	Primary cortical neurons	530 nm, 6 mW/cm^2	→ Viability (fluorescein diacetate and propidium iodide staining)	[10]
	~ 60 nm, porous	Human umbilical vein endothelial cells	530 nm, 6 mW/cm^2	→ Viability (Alamar blue assay)	[22]
	~ 200 nm, PEDOT:PSS multicores within P3HT	Human umbilical vein endothelial cells	Dark	→ Proliferation (Alamar blue assay)	[23]
	~ 60 nm P3HT nanoparticles in hydrogel	Mouse glioma 261 cells	467 nm, 40 – 60 mW/cm^2	↓ Viability (flow cytometry)	[24]
	~ 100 nm P3HT nanoparticles in hydrogel	Human neuroblastoma cells (SH-SY5Y)	Dark	↑ Viability (Ready probes cell viability imaging kit)	[27]
	~ 148 nm and ~ 344 nm	Human embryonic kidney 293 cells	Dark	↓ Proliferation (MTT assay)	[36]
	~ 237 nm	Human embryonic kidney 293 cells	Dark	↓ Viability (MTT assay)	[37]
	~280 nm	Mouse brain capillary endothelial cells (bEND.3)	White light, 1.3 mW/cm^2	↓ Viability (MTT assay)	This work
	~170 nm, P3HT:PCBM nanoparticles	Mouse brain capillary endothelial cells (bEND.3)	White light, 1.3 mW/cm^2	↓ Viability (MTT assay)	This work

S2. Thickness of the poly(3-hexylthiophene-2,5-diyl) (P3HT) films presented to bEnd.3 cells

The thickness of the P3HT films presented to the bEnd.3 cells was assessed using atomic force microscopy (AFM). Films were deposited onto glass microscope coverslips as described in the main text. To prepare samples for AFM analysis, the polymer surface was scratched with a scalpel, creating a boundary between uncoated glass and P3HT-coated regions. This interface allowed direct measurement of film height. Representative AFM results are shown in Figure S1. As also mentioned in the main text, the average film thickness obtained via this procedure was 82.4 ± 2.2 nm.

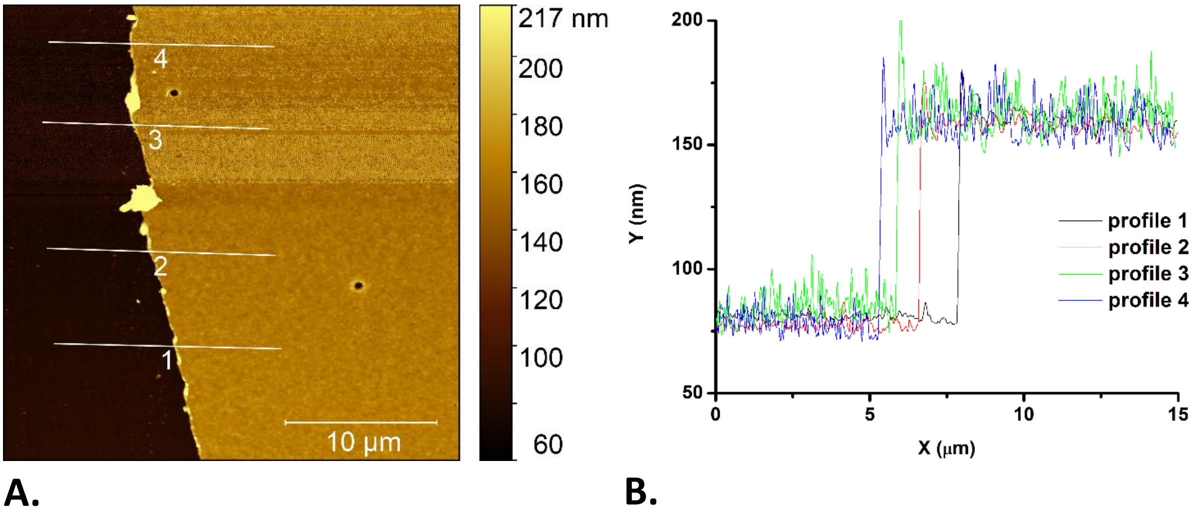

Figure S1. *AFM image of a boundary between uncoated glass and P3HT-coated glass* ***(A)*** *and profiles extracted from the image of the boundary* ***(B)****.*

S3. AFM images of the P3HT-based nanoparticles presented to the bEnd.3 cells

AFM images of the P3HT-based nanoparticles used in our experiments with bEnd.3 cells are shown in Figure 2A and Figure 3A of the main text. Figure S2 below presents additional AFM images of the P3HT nanoparticles (panel A) and of the P3HT-PCBM nanoparticles (panel B). (Observation: PCBM stays for [6,6]-phenyl-C61-butyric acid methyl ester.) These images confirm the spherical morphology and size range of the particles as described in the main text.

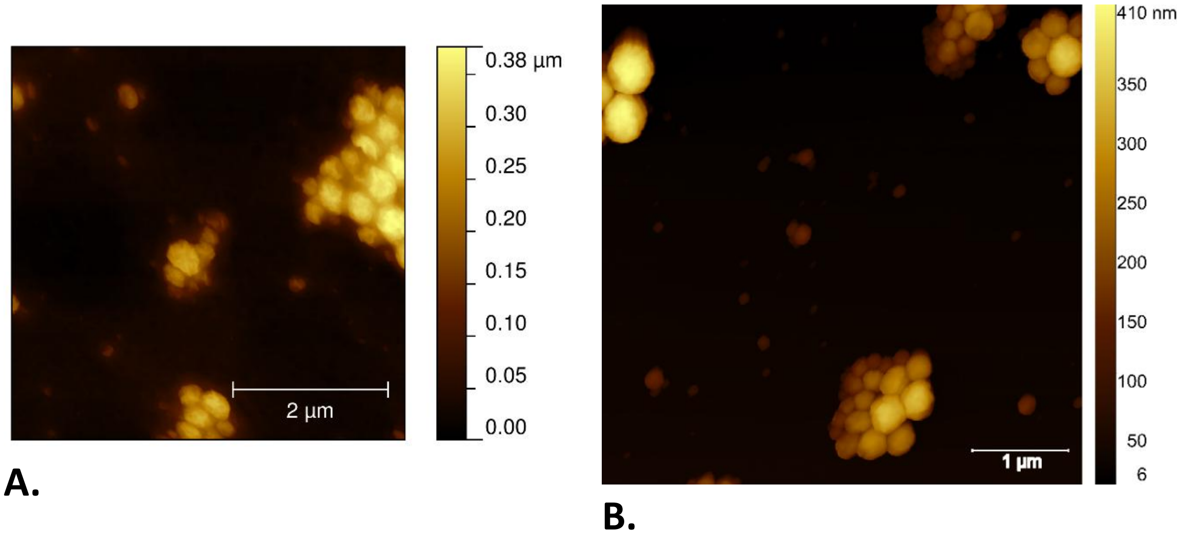

Figure S2. *AFM images of P3HT nanoparticles* ***(A)*** *and of P3HT-PCBM nanoparticles* ***(B)****. Observations: The AFM images shown here were obtained in air, with the nanoparticles deposited onto a glass slide. The aggregates visible in these images formed during the evaporation of water from the nanoparticle suspension drop casted on the glass slide. The nanoparticle suspension itself is stable, without pronounced signs of aggregation or sedimentation.*

S4. Schematic representation of the timeline of the biocompatibility tests

Figure S3 schematically presents the timeline of the biocompatibility tests conducted on P3HT films, P3HT nanoparticles and P3HT-PCBM nanoparticles. Important to note, the testing procedure was slightly adjusted for nanoparticles compared to that used for the films.

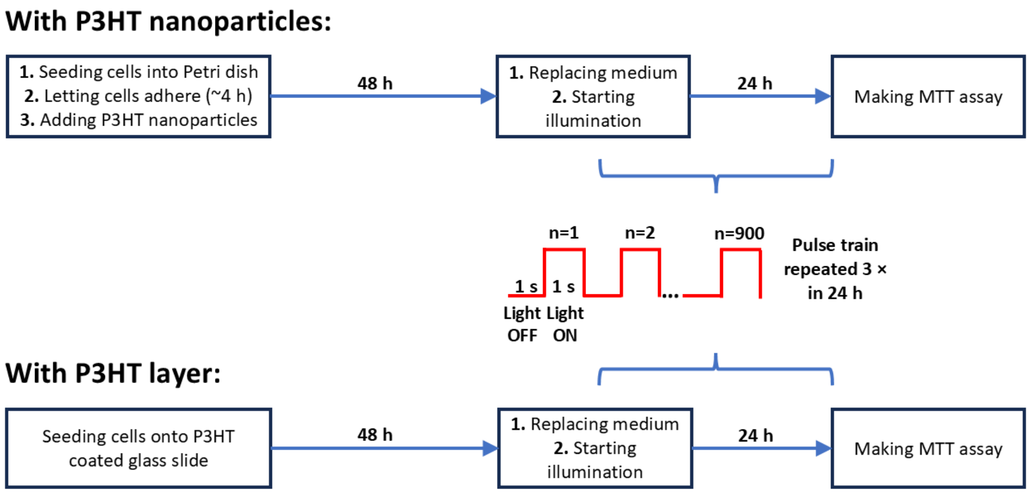

Figure S3. *Schematic representation of the timeline of the biocompatibility tests conducted on P3HT films, P3HT nanoparticles and P3HT-PCBM nanoparticles.*

S5. Estimating the concentration of nanoparticles from absorbance data

The nanoparticle concentration was calculated from the absorbance of the suspension using the Beer–Lambert law: $A = \varepsilon \times c \times L$ (where A is the absorbance, ε is the molar extinction coefficient in L/mol × cm, c is the concentration in mol/L, and L is the path length in cm). By substituting $A = 0.5$ (measured at 570 nm), $\varepsilon = 4.86 \times 106$ L/mol × cm (per polymer chain), and $L = 1$ cm, we obtain $c = 1.03 \times 10^{-7}$ mol/L. Accounting for the molecular weight of our polymer (Mw = 74000 Da) converts this to 7.6 µg/mL of P3HT in our experiments at cellular level. Important to note that, while A, L and Mw can be determined experimentally, ε itself had to be calculated. We applied the Beer–Lambert law once again to an 80 nm thick P3HT film characterized by an absorbance of 0.578 (at 570 nm) and a density of 1.1 g/cm^3 (according to literature [61–64]) that translates to a P3HT concentration of 0.0149 mol/L. Because the optical properties of the P3HT films and nanoparticles are not perfectly matched, the calculated P3HT concentration (i.e., 7.6 µg/mL) should be viewed as an approximation rather than an exact value.

S6. Estimating the localization of P3HT-based nanoparticles at cellular level from dark-field microscopy images

In addition to Figures 6 and 7 from the main text (which show dark-field microscopy images of cells incubated with P3HT-based nanoparticles), Figure S4 shows enlarged images of bEND.3 cells exposed to P3HT-PCBM nanoparticles. Careful inspection of these images reveals a size-dependent localization pattern: smaller nanoparticles are predominantly observed in the intracellular space around the nucleus, whereas larger nanoparticles are mainly detected at the cell surface, often at a greater distance from the nucleus.

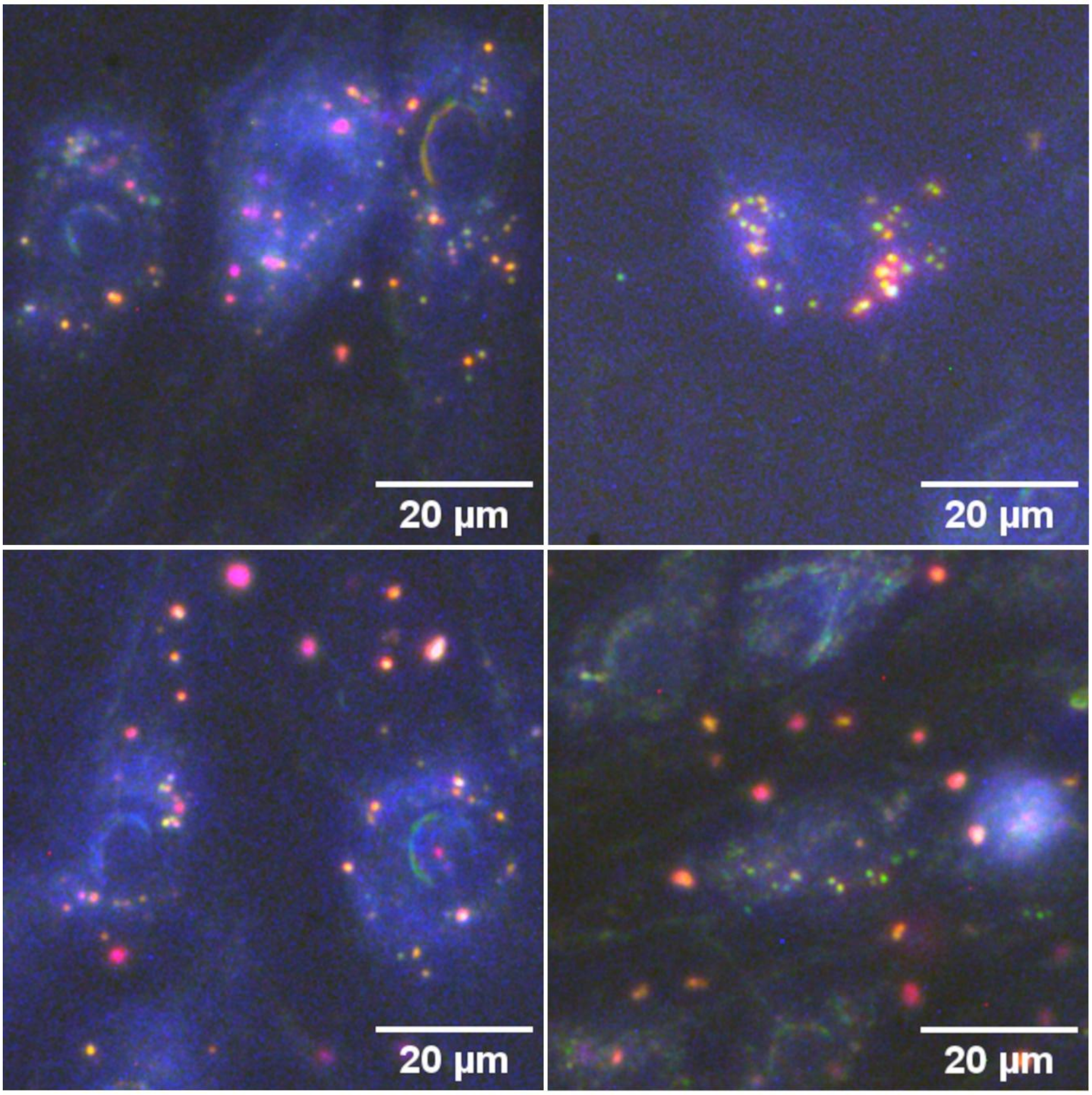

Figure S4. *Representative dark-field microscopy images of bEND.3 cells incubated with P3HT-PCBM nanoparticles. The images illustrate that the nanoparticles (brighter spots) are localized both at the cell surface (see the larger nanoparticles usually found far from the circular nucleus) and within the cytoplasm (see the smaller nanoparticles distributed around the perinuclear region as they cannot cross the nuclear membrane). Observation: The blue channel (in which the nanoparticles are not visible) was adjusted for brightness and contrast before being merged with the green channel and the red channel (in which the nanoparticles are visible). This manipulation enhanced somewhat the visibility of the cytoplasm in the above images.*

S7. Viability values measured while testing the compatibility of the P3HT-based materials with bEnd.3 cells

Table S2 displays the viability values measured while testing the compatibility of P3HT films with bEnd.3 cells. These data were used to generate Figure 5E presented in the main text.

Table S2. *Viability values measured while testing the compatibility of P3HT films with bEnd.3 cells.*

**Experimental group**	**Observed viability**								
	**Polymer batch 1**			**Polymer batch 2**			**Polymer batch 3**		
	**Exp. 1**	**Exp. 2**	**Exp. 3**	**Exp. 4**	**Exp. 5**	**Exp. 6**	**Exp. 7**	**Exp. 8**	**Exp. 9**
**- P3HT film / - light**	100.7	103.1	96.1	96.1	98.0	105.8	90.9	103.8	105.1
**- P3HT film / + light**	95.2	101.6	90.1	158.6	149.4	108.5	95.6	85.7	87.2
**+ P3HT film / - light**	106.2	97.5	110.1	107.8	109.3	116.3	105.9	100.4	104.5
**+ P3HT film / + light**	122.1	133.8	148.0	103.1	97.6	106.0	113.4	104.8	105.6

Table S3 displays the viability values measured while testing the compatibility of P3HT nanoparticles with bEnd.3 cells. These data were used to generate Figure 6E presented in the main text.

Table S3. *Viability values measured while testing the compatibility of P3HT nanoparticles (P3HT NPs) with bEnd.3 cells.*

**Experimental group**	**Observed viability**								
	**P3HT NPs batch 1**			**P3HT NPs batch 2**			**P3HT NPs batch 3**		
	**Exp. 1**	**Exp. 2**	**Exp. 3**	**Exp. 4**	**Exp. 5**	**Exp. 6**	**Exp. 7**	**Exp. 8**	**Exp. 9**
**- P3HT NPs / - light**	85.5	107.6	106.8	96.5	104.3	99.0	94.2	100.9	104.7
**- P3HT NPs / + light**	100.9	102.0	115.1	81.9	73.9	74.2	115.5	118.1	117.3
**+ P3HT NPs / - light**	78.7	77.6	82.6	72.2	64.0	n.a.	72.7	70.5	59.1
**+ P3HT NPs / + light**	78.3	73.1	86.2	70.0	70.9	64.5	57.0	75.2	76.1

Table S4 displays the viability values measured while testing the compatibility of P3HT-PCBM nanoparticles with bEnd.3 cells. These data were used to generate Figure 7E presented in the main text.

Table S4. *Viability values measured while testing the compatibility of P3HT-PCBM nanoparticles (P3HT-PCBM NPs) with bEnd.3 cells.*

**Experimental group**	**Observed viability**								
	**P3HT-PCBM NPs batch 1**			**P3HT-PCBM NPs batch 2**			**P3HT-PCBM NPs batch 3**		
	**Exp. 1**	**Exp. 2**	**Exp. 3**	**Exp. 4**	**Exp. 5**	**Exp. 6**	**Exp. 7**	**Exp. 8**	**Exp. 9**
**- P3HT-PCBM NPs / - light**	86.7	99.4	113.8	89.9	98.8	111.2	92.7	108.1	99.1
**- P3HT-PCBM NPs / + light**	107.1	83.5	81.3	118.6	107.2	106.2	108.1	102.7	103.5
**+ P3HT-PCBM NPs / - light**	68.4	63.5	n.a.	92.7	93.5	89.3	80.8	78.4	79.1
**+ P3HT-PCBM NPs / + light**	70.0	55.5	53.2	91.0	91.0	96.8	82.9	74.9	72.6

S8. References

(Observation: The reference numbering from the main text has been maintained in the Supplementary Material.)

4. Aziz, I.A.; Maver, L.; Giannasi, C.; Niada, S.; T. Brini, A.; Rosa Antognazza, M. Polythiophene-Mediated Light Modulation of Membrane Potential and Calcium Signalling in Human Adipose-Derived Stem/Stromal Cells. *J. Mater. Chem. C* **2022**, *10*, 9823–9833. https://doi.org/10.1039/D2TC01426B.
5. Campione, P.; Rizzo, M.G.; Bauso, L.V.; Ielo, I.; Messina, G.M.L.; Calabrese, G. Osteoblastic Differentiation of Human Adipose-Derived Mesenchymal Stem Cells on P3HT Thin Polymer Film. *J. Funct. Biomater.* **2025**, *16*, 10. https://doi.org/10.3390/jfb16010010.
6. Lodola, F.; Rosti, V.; Tullii, G.; Desii, A.; Tapella, L.; Catarsi, P.; Lim, D.; Moccia, F.; Antognazza, M.R. Conjugated Polymers Optically Regulate the Fate of Endothelial Colony-Forming Cells. *Sci. Adv.* **2019**, *5*, eaav4620. https://doi.org/10.1126/sciadv.aav4620.
10. Wu, C.; Pu, Y.; Zhang, Y.; Liu, X.; Qiao, Z.; Xin, N.; Zhou, T.; Chen, S.; Zeng, M.; Tang, J.; et al. A Bioactive and Photoresponsive Platform for Wireless Electrical Stimulation to Promote Neurogenesis. *Adv. Healthc. Mater.* **2022**, *11*, 2201255. https://doi.org/10.1002/adhm.202201255.
13. DiFrancesco, M.L.; Colombo, E.; Papaleo, E.D.; Maya-Vetencourt, J.F.; Manfredi, G.; Lanzani, G.; Benfenati, F. A Hybrid P3HT-Graphene Interface for Efficient Photostimulation of Neurons. *Carbon* **2020**, *162*, 308–317. https://doi.org/10.1016/j.carbon.2020.02.043.
15. Lodola, F.; Martino, N.; Tullii, G.; Lanzani, G.; Antognazza, M.R. Conjugated Polymers Mediate Effective Activation of the Mammalian Ion Channel Transient Receptor Potential Vanilloid 1. *Sci. Rep.* **2017**, *7*, 8477. https://doi.org/10.1038/s41598-017-08541-6.
21. Criado-Gonzalez, M.; Bondi, L.; Marzuoli, C.; Gutierrez-Fernandez, E.; Tullii, G.; Ronchi, C.; Gabirondo, E.; Sardon, H.; Rapino, S.; Malferrari, M.; et al. Semiconducting Polymer Nanoporous Thin Films as a Tool to Regulate Intracellular ROS Balance in Endothelial Cells. *ACS Appl. Mater. Interfaces* **2023**, *15*, 35973–35985. https://doi.org/10.1021/acsami.3c06633.
22. Criado-Gonzalez, M.; Marzuoli, C.; Bondi, L.; Gutierrez-Fernandez, E.; Tullii, G.; Lagonegro, P.; Sanz, O.; Cramer, T.; Antognazza, M.R.; Mecerreyes, D. Porous Semiconducting Polymer Nanoparticles as Intracellular Biophotonic Mediators to Modulate the Reactive Oxygen Species Balance. *Nano Lett.* **2024**, 24, 7244-7251. https://doi.org/10.1021/acs.nanolett.4c01195.
23. Tullii, G.; Bellacanzone, C.; Comas Rojas, H.; Fumagalli, F.; Ronchi, C.; Villano, A.; Gobbo, F.; Bogar, M.; Sartori, B.; Sassi, P.; et al. Composite Thiophene-Based Nanoparticles: Revisiting the PEDOT:PSS/P3HT Interface for Living-Cell Optical Modulation. *ACS Appl. Mater. Interfaces* **2025**, 17, 22434-22447. https://doi.org/10.1021/acsami.5c02115.
24. Natera Abalos, R.; Abdel Aziz, I.; Caverzan, M.; Sosa Lochedino, A.; Ibarra, L.E.; Gallastegui, A.; Chesta, C.A.; Lorena Gómez, M.; Mecerreyes, D.; Palacios, R.E.; et al. Poly(3-Hexylthiophene) Nanoparticles as Visible-Light Photoinitiators and Photosensitizers in 3D Printable Acrylic Hydrogels for Photodynamic Therapies. *Mater. Horiz.* **2025**, 12, 2524-2534. https://doi.org/10.1039/D4MH01802H.
27. Ciocca, M.; Febo, C.; Gentile, G.; Orlando, A.; Massoumi, F.; Altana, A.; Cantarella, G.; Zanon, A.; Gaiardo, A.; Lugli, P.; et al. 3D-Bioprinted Light-Sensitive Cell Scaffold Based on Alginate-Conjugated Polymer Nanoparticles for Biophotonics Applications. *BioNanoScience* **2025**, *15*, 251. https://doi.org/10.1007/s12668-025-01863-0.

28. Campione, P.; Latte Bovio, C.; Calabrese, G.; Santoro, F.; Messina, G.M.L. P3HT-Based Electroactive Films for In Vitro Neuronal Cell Interfacing. *Adv. Mater. Interfaces* **2025**, 12, 2400776. https://doi.org/10.1002/admi.202400776.
30. Martino, N.; Feyen, P.; Porro, M.; Bossio, C.; Zucchetti, E.; Ghezzi, D.; Benfenati, F.; Lanzani, G.; Antognazza, M.R. Photothermal Cellular Stimulation in Functional Bio-Polymer Interfaces. *Sci. Rep.* **2015**, *5*, 8911. https://doi.org/10.1038/srep08911.
32. Benfenati, V.; Martino, N.; Antognazza, M.R.; Pistone, A.; Toffanin, S.; Ferroni, S.; Lanzani, G.; Muccini, M. Photostimulation of Whole-Cell Conductance in Primary Rat Neocortical Astrocytes Mediated by Organic Semiconducting Thin Films. *Adv. Healthc. Mater.* **2014**, *3*, 392–399. https://doi.org/10.1002/adhm.201300179.
33. Scarpa, G.; Idzko, A.-L.; Götz, S.; Thalhammer, S. Biocompatibility Studies of Functionalized Regioregular Poly(3-Hexylthiophene) Layers for Sensing Applications. *Macromol. Biosci.* **2010**, *10*, 378–383. https://doi.org/10.1002/mabi.200900412.
34. Šafaříková, E.; Švihálková Šindlerová, L.; Stříteský, S.; Kubala, L.; Vala, M.; Weiter, M.; Víteček, J. Evaluation and Improvement of Organic Semiconductors' Biocompatibility towards Fibroblasts and Cardiomyocytes. *Sens. Actuators B Chem.* **2018**, *260*, 418–425. https://doi.org/10.1016/j.snb.2017.12.108.
36. Zucchetti, E.; Zangoli, M.; Bargigia, I.; Bossio, C.; Maria, F.D.; Barbarella, G.; D'Andrea, C.; Lanzani, G.; Antognazza, M.R. Poly(3-Hexylthiophene) Nanoparticles for Biophotonics: Study of the Mutual Interaction with Living Cells. *J. Mater. Chem. B* **2017**, *5*, 565–574. https://doi.org/10.1039/C6TB02047J.
37. Bossio, C.; Abdel Aziz, I.; Tullii, G.; Zucchetti, E.; Debellis, D.; Zangoli, M.; Di Maria, F.; Lanzani, G.; Antognazza, M.R. Photocatalytic Activity of Polymer Nanoparticles Modulates Intracellular Calcium Dynamics and Reactive Oxygen Species in HEK-293 Cells. *Front. Bioeng. Biotechnol.* **2018**, *6*, 114. https://doi.org/10.3389/fbioe.2018.00114.
61. Bounioux, C.; Díaz-Chao, P.; Campoy-Quiles, M.; Martín-González, M.S.; Goñi, A.R.; Yerushalmi-Rozen, R.; Müller, C. Thermoelectric Composites of Poly(3-Hexylthiophene) and Carbon Nanotubes with a Large Power Factor. *Energy Environ. Sci.* **2013**, *6*, 918–925. https://doi.org/10.1039/C2EE23406H.
62. Untilova, V.; Hynynen, J.; Hofmann, A.I.; Scheunemann, D.; Zhang, Y.; Barlow, S.; Kemerink, M.; Marder, S.R.; Biniek, L.; Müller, C.; et al. High Thermoelectric Power Factor of Poly(3-Hexylthiophene) through In-Plane Alignment and Doping with a Molybdenum Dithiolene Complex. *Macromolecules* **2020**, *53*, 6314–6321. https://doi.org/10.1021/acs.macromol.0c01223.
63. Lee, Y.; Mongare, A.; Plant, A.; Ryu, D. Strain–Microstructure–Optoelectronic Inter-Relationship toward Engineering Mechano-Optoelectronic Conjugated Polymer Thin Films. *Polymers* **2021**, *13*, 935. https://doi.org/10.3390/polym13060935.
64. Casalegno, M.; Famulari, A.; Meille, S.V. Modeling of Poly(3-Hexylthiophene) and Its Oligomer's Structure and Thermal Behavior with Different Force Fields: Insights into the Phase Transitions of Semiconducting Polymers. *Macromolecules* **2022**, *55*, 2398–2412. https://doi.org/10.1021/acs.macromol.2c00131.
